# Targeted Antibiotic Prophylaxis in Percutaneous Nephrolithotomy: Results of a Protocol Based on Preoperative Urine Culture and Risk Assessment

**DOI:** 10.3390/jcm14155249

**Published:** 2025-07-24

**Authors:** Felipe Osorio-Ospina, Gonzalo Bueno-Serrano, María Pilar Alcoba-García, Juan Tabares-Jiménez, Blanca Gómez-Jordana-Mañas, Elena García-Criado, Joaquin Ruiz-de-Castroviejo, Xabier Pérez-Aizpurua, Jaime Jorge Tufet-I-Jaumot, Raúl González-Páez, Jose Carlos Matta-Pérez, Beatriz Yanes-Glaentzlin, Juan Francisco Jiménez-Abad, José Maria Alcázar Peral, Nerea Carrasco Antón, Elizabet Petkova-Saiz, Carmen González-Enguita

**Affiliations:** 1 Department of Urology, Hospital Universitario Fundación Jiménez Díaz, 28040 Madrid, Spain; felipe.osorio@quironsalud.es (F.O.-O.); pilar.alcoba@quironsalud.es (M.P.A.-G.); jtabares@quironsalud.es (J.T.-J.); blanca.gomezj@quironsalud.es (B.G.-J.-M.); elena.gcriado@quironsalud.es (E.G.-C.); xabier.perez@quironsalud.es (X.P.-A.); jaime.tufet@quironsalud.es (J.J.T.-I.-J.); raul.gpaez@quironsalud.es (R.G.-P.); jose.matta@quironsalud.es (J.C.M.-P.); beatriz.yanes@quironsalud.es (B.Y.-G.); juan.jabad@quironsalud.es (J.F.J.-A.); cgenguita@fjd.es (C.G.-E.); 2 Quirónsalud España, 28006 Madrid, Spain; jose.alcazar@quironsalud.es; 3 Department of Infectious Diseases, Clínica Universitaria de Navarra, 28027 Madrid, Spain; ncarrascoa@unav.es; 4 Department of Infectious Diseases, Hospital Universitario Fundación Jiménez Díaz, 28040 Madrid, Spain; epetkovas@fjd.es

**Keywords:** percutaneous nephrolithotomy, antibiotic prophylaxis, urinary tract infection, urine culture, postoperative sepsis

## Abstract

**Background:** Infectious complications are common after percutaneous nephrolithotomy (PCNL). Clinical guidelines recommend, previous to surgery, prolonged antibiotic regimens in patients with preoperative positive urine cultures to reduce infectious risk. However, such strategies may increase selective pressure and promote antimicrobial resistance. Evidence supporting the use of a single antibiotic dose tailored to culture sensitivity in these cases is limited but emerging. **Methods:** We conducted a retrospective observational study including 187 PCNL procedures performed between 2021 and 2023 under an individualized antibiotic prophylaxis protocol. Patients with negative or contaminated urine cultures received a single empirical dose, while those with recent positive cultures received a single dose based on antimicrobial susceptibility testing. Postoperative complications—including fever, sepsis, and a composite outcome—were analyzed through multivariable logistic regression, comparing high- and low-risk patients. **Results:** A total of 67.9% of procedures were performed in patients meeting at least one high-risk criterion, including a positive preoperative urine culture in 32.1%. The overall incidence of infectious complications was 11.9% (fever 8.7%, sepsis 3.2%), with no significant differences between risk groups. A low concordance was observed between preoperative and intraoperative urine cultures (Spearman = 0.3954). **Conclusions:** A single preoperative antibiotic dose adjusted to the antibiogram, even in patients with a positive urine culture, was not associated with increased infectious complications. This approach is an initial step that supports a rational and individualized prophylactic strategy aligned with the goals of antimicrobial stewardship programs (ASPs).

## 1. Introduction

Urinary stone disease is highly prevalent, with an estimated occurrence of 4.2% in the Spanish population, showing a male-to-female ratio of 2:1 [1]. Its clinical relevance lies not only in its frequency but also in its high recurrence rate, which approaches 39% at 15 years [2].

Percutaneous nephrolithotomy (PCNL) is the treatment of choice for large stones (>1.5–2 cm) and represents a major advancement over open surgery, offering reduced complication rates and faster recovery [3].

Despite its benefits, infectious complications remain the most frequent adverse events following PCNL, affecting up to 10% of patients, implying a clinical burden in terms of patient morbidity and healthcare resource utilization. Several studies have linked these complications to prolonged hospital stays, increased costs, and, in severe cases, intensive care unit admission and mortality [4,5,6,7,8].

These risks are further magnified by the growing prevalence of multidrug-resistant organisms, which complicate empirical prophylaxis and are magnified by inappropriate or excessive antibiotic exposure [7,8,9,10].

Consequently, there is a clear need to refine perioperative prophylactic strategies in urology particularly by following the antimicrobial stewardship programs (ASPs), tailoring antibiotic prophylaxis to individual risk profiles and microbiological data whenever possible, as an effective way to minimize resistance without compromising patient safety [7,10,11].

Even with current recommendations, postoperative sepsis occurs in 0.3% to 7.6% of cases and may reach a mortality rate of 66% in severe presentations [6]. This has driven the development of preventive strategies centered on rational antibiotic use to reduce ecological impact and the rise of antimicrobial resistance.

PCNL is considered a clean-contaminated surgical procedure. Current guidelines recommend a single prophylactic antibiotic dose in patients with sterile urine, adjusted to local resistance patterns, or a 3–5 day antibiotic regimen in those with positive preoperative urine cultures [12,13].

It is important to note that prophylactic antibiotics must be administered correctly. Current recommendations indicate that the antibiotic should be given within 120 min prior to surgical incision; however, this interval may vary depending on the specific agent used. For example, beta-lactams with short half-lives are preferably administered within 60 min before incision, whereas aminoglycosides should ideally be administered within 90 min prior to the procedure [12].

Therefore, the aim of this study was to evaluate the results of a targeted prophylactic protocol tailored to the patient’s clinical and microbiological profile in patients undergoing PCNL, including those with positive preoperative urine cultures.

## 2. Materials and Methods

### 2.1. Protocol Development

The individualized prophylaxis protocol was developed in collaboration with the infectious diseases department, following institutional antimicrobial stewardship policies. Its primary aim was to optimize antibiotic use by reducing unnecessary exposure while maintaining adequate infection prevention. The decision to administer a single dose, even in high-risk patients, was supported by local susceptibility patterns and national recommendations from the Spanish Society of Infectious Diseases and Clinical Microbiology (SEIMC) and the Spanish Association of Surgeons (AEC) [12].

### 2.2. Patients

A retrospective descriptive study was conducted on patients undergoing PCNL at our institution between January 2021 and December 2023 under an individualized antibiotic prophylaxis protocol.

We collected clinical data, stone characteristics, preoperative urine culture and renal puncture culture results, presence of urinary drainage, and postoperative complications, particularly infectious ones such as fever, bacteremia, or sepsis (defined as Sequential Organ Failure Assessment (SOFA) score ≥ 2 or positive blood cultures). Each procedure was analyzed independently, including those with multiple surgeries.

### 2.3. Risk Assessment

High infectious risk was defined based on AUA criteria: positive urine culture, indwelling catheter or nephrostomy, staghorn stone (Guy’s IV classification), and/or diabetes mellitus [14].

### 2.4. Antibiotic Strategy

Patients with negative or no recent cultures received a single dose of cefuroxime (or amikacin for beta-lactam allergies). In cases with recent positive urine cultures (within one week, or up to three months if asymptomatic), a single dose adjusted to the antibiogram was administered. The prophylactic antibiotic was administered 30 min before the start of surgery.

### 2.5. Urine Cultures

Preoperative spontaneous urine culture was obtained.

A renal puncture urine sample was collected intraoperatively through the percutaneous tract at the beginning of the procedure, whenever technically feasible and appropriate sterile containers were available. These samples were used to compare renal urine microbiology with the preoperative bladder urine culture. The rationale for this comparison was to assess concordance between both sites, as discrepancies may suggest that bladder urine is an unreliable surrogate for renal infection.

Patients without available urine cultures or with contaminated results received empirical prophylaxis but were excluded from multivariable analysis. Also excluded were those with symptomatic UTI, purulent urine during surgery, or a prior history of sepsis after endourological procedures. In all of these cases, conventional therapeutic antibiotics were initiated perioperatively.

### 2.6. Surgical Approach

All PCNLs were performed in the supine position, under general anesthesia and by the same surgical team. A single-tract access was obtained using an 18G needle under fluoroscopic guidance, followed by dilation using the MIP system. Stone fragmentation was carried out using laser lithotripsy or pneumatic lithotripter, and fragments were extracted using suction or a stone basket.

The maximum surgical time for urological intervention was limited to 120 min; longer durations occurred in specific cases deemed clinically appropriate.

### 2.7. Inpatient Follow-Up

All patients were monitored postoperatively. Vital signs, laboratory parameters, including white blood cell count and C-reactive protein, were assessed the first postoperative day.

Blood cultures were obtained in patients presenting fever ≥ 38 °C or clinical signs of systemic infection. Infectious complications were classified according to the Clavien–Dindo system when applicable.

### 2.8. Statistical Analysis

Patients were excluded from the protocol and also from analysis if they presented with an active symptomatic urinary tract infection, pyuria or purulent urine during renal puncture, or a documented episode of sepsis prior to the procedure. Additionally, cases with unavailable or contaminated preoperative urine cultures were excluded from the multivariable analysis to preserve consistency and avoid potential bias.

Statistical analysis was performed using SPSS v30 (IBM SPSS, Armonk, NY, USA). Quantitative variables were described as median and interquartile range and compared using the Kruskal–Wallis test. Categorical variables were expressed as frequencies and percentages and compared using chi-square or Fisher’s exact test. Multivariable analysis was performed using logistic regression, reporting odds ratios (ORs) with 95% confidence intervals (CIs) and *p*-values.

## 3. Results

A total of 170 patients undergoing 187 PCNL procedures were included. Baseline characteristics are summarized in Table 1.

Preoperative urine cultures were available in 172 cases (92%), with 58.1% (100/172) sterile, 34.8% (60/172) positive, and 7% (12/172) contaminated. Renal puncture cultures were obtained in 121 procedures (64.7%), with results as follows: 78.5% sterile (95/121), 19.8% positive (24/121), and 1.7% contaminated (2/121).

Among patients with positive preoperative urine cultures, Escherichia coli was the most frequently isolated microorganism (37.7%), followed by Proteus spp. (18.0%) and Klebsiella spp. (11.5%). These findings are consistent with previous literature describing E. coli as the dominant pathogen in urinary stone formers. In renal puncture cultures, however, the distribution varied, with E. coli representing 37.5% and Proteus spp. (29.2%) accounting for a higher proportion. Other less common organisms such as *Aerococcus*, *Citrobacter koseri*, *Candida albicans*, and mixed anaerobic flora were also identified. Microbiological isolates are detailed in Table 2.

Of the 85 procedures with at least one positive culture, 72 (84.7%) showed discordant results between preoperative and intraoperative samples. This discordance included either mismatched isolates or the presence of a sterile intraoperative culture despite a previously positive preoperative sample. The most frequent mismatches involved Proteus spp. and Klebsiella spp., while isolates such as Enterococcus spp. and mixed anaerobic flora were inconsistently recovered. These findings highlight the limited reliability of bladder urine cultures in predicting upper tract colonization and support the potential clinical utility of renal puncture cultures in selected patients.

To further assess the degree of discordance between preoperative and intraoperative urine cultures, a correlation analysis was performed using Spearman’s rank coefficient. This revealed only a moderate correlation (ρ = 0.3954), reinforcing the notion that bladder urine does not reliably reflect renal colonization.

High infectious risk criteria were present in 67.9% (127/187) of procedures: prior urinary drainage (39.6%), positive urine culture (32.1%), staghorn calculi (18.7%), or diabetes mellitus (17.1%).

The median operative time was 100 min (IQR 35–180), with 34.2% of procedures exceeding 120 min. Although the institutional protocol defined 120 min as the upper surgical time limit, these exceptions were clinically justified. No statistical or clinical differences were observed between longer operative time and the incidence of infectious complications in our cohort (*p* = 0.534), although this is a well-known risk factor, particularly in high-risk patients.

Infectious postoperative complications occurred in 11.9% of procedures in the high infectious risk group, most commonly fever (8.7%), with sepsis being diagnosed in 3.2%. Meanwhile, in the low infectious risk group, we found a fever rate of 4.3%, and no sepsis events were reported.

Readmission within 30 days occurred in 5% of patients in the low-risk group and 4.7% in the high-risk group, with no statistically significant difference between groups (*p* = 0.934). No differences were observed when stratifying by preoperative urine culture alone (*p* = 0.454). Most readmissions were due to febrile episodes, pain, or hematuria, and these were managed conservatively.

Multivariable analysis was performed in the high-risk infectious risk group to examine predictors of fever, sepsis, and the composite outcome after PCNL, including variables such as positive urine culture, diabetes mellitus, prior urinary drainage, and Guy’s IV classification defined as a complete staghorn calculus. No statistically significant differences were found in postoperative fever or sepsis between low- and high-risk groups (*p* = 0.321). Adjusted ORs and 95% CIs are shown in Table 3.

The median hospital stay in low-risk patients was 25 h in comparation with 32 h for high-risk patients, without a clinically significant difference.

## 4. Discussion

Infectious complications remain a leading cause of morbidity and mortality after stone surgery, particularly following PCNL. Our cohort showed rates of postoperative fever and sepsis (8.7% and 3.2%, respectively) consistent with those reported in the literature for patients with positive urine cultures, although existing studies often focus on negative-culture populations and report highly variable outcomes (0.3%-7.6 for sepsis and up to 32% for fever) [4,9,15,16].

Several randomized trials and observational studies have evaluated prophylactic antibiotic strategies in patients undergoing PCNL, with a particular focus on high-risk subgroups. Sur et al. found that extending antibiotic prophylaxis for five days prior to PCNL in moderate- to high-risk patients reduced infectious complications compared 7 to 2 days antibiotic course; however, a bacteriostatic antibiotic was used instead of bactericidal effects of the antibiotic used in our protocol [17]. Also, this approach may contribute to increased antimicrobial exposure and resistance.

In contrast, Chew et al. demonstrated that in low-risk patients, single-dose antibiotic prophylaxis is sufficient to reduce infectious events, consistent with our findings supporting the use of a single dose in selected patients [4,18]. However, its use in high-risk populations remains controversial. Current guidelines recommend treating patients with a positive urine culture or clinically significant urinary tract infections with a course of antibiotics for at least 5 days prior to surgery, ideally guided by susceptibility testing. Surgical intervention should be delayed until the infection is clinically resolved and, when possible, supported by a negative urine culture [17,19,20].

Some authors, such as Potretzke, have proposed that extending preoperative antibiotics to 7 days may reduce sepsis in high-risk patients compared to shorter regimens, even with negative cultures. However, these findings lack generalizability when the population is not stratified by risk [10,11].

Our results support single-dose prophylaxis, even in high-risk patients, as an effective strategy to prevent infectious complications. This aligns with guidelines from the Spanish Society of Infectious Diseases and Clinical Microbiology (SEIMC) and the Spanish Association of Surgeons (AEC), which classify PCNL as a clean-contaminated procedure and recommend a single preoperative antibiotic dose (recommendation A-I) [12,21].

Similarly, the European Association of Urology (EAU) supports single-dose prophylaxis even in patients with infectious risk factors, contributing to reduced antimicrobial pressure [13,22].

Moreover, prolonged antibiotic prophylaxis has not been shown to improve clinical outcomes and may increase the risk of adverse events, including *Clostridium difficile* infection, antimicrobial resistance, and acute kidney injury. Our findings are consistent with the principles of antimicrobial stewardship programs (ASPs), which promote minimizing unnecessary antibiotic exposure to reduce resistance and optimize outcomes in urological surgery [12].

Although asymptomatic bacteriuria is a known risk factor for infection, we found no association between positive urine cultures and the development of fever or sepsis, with rates in line with published data. Some authors, like Süelözgen, have also reported that preoperative positive cultures do not correlate with higher complication rates [23,24].

Perioperative antibiotic selection in our clinical setting should provide coverage against Gram-negative enteric bacteria and *Enterococcus* spp., typically using first- or second-generation cephalosporins or trimethoprim/sulfamethoxazole. This recommendation is supported by our local antimicrobial susceptibility profile, which shows favorable sensitivity to these agents. For enterococcal infections, penicillin or ampicillin remain first-line treatments, while aminoglycosides and cephalosporins are effective against *Proteus*, a species frequently associated with stone disease [25].

The implementation of a single dose, risk-adapted prophylaxis protocol at our institution shows feasibility and clinical safety, even in high-risk patients. By tailoring antibiotic selection to preoperative urine culture results and avoiding unnecessary prolonged regimens, this approach aligns with antimicrobial stewardship goals while maintaining low infection rates. From a clinical management perspective, the protocol has standardized preoperative assessment and facilitated multidisciplinary coordination between urology and infectious diseases. In our experience, this strategy has not led to an increase in infectious complications and has not required escalation of postoperative antibiotics in most cases.

Moreover, the reduction in antibiotic exposure may contribute to lower rates of drug-related adverse events, including nephrotoxicity and *Clostridium difficile* infection, although this was not formally evaluated in this study.

Thus, the practical applicability of this protocol suggests that it could serve as a model for other centers aiming to balance infection prevention with responsible antibiotic use.

Despite appropriate prophylaxis, postoperative fever may still occur in patients with negative preoperative urine cultures, as calculi can harbor bacteria that evade detection. For this reason, obtaining intraoperative cultures—either from the renal pelvis or directly from the stone—is recommended and has been identified as a predictor of infectious complications. Moreover, our data showed a high rate of discordance among the 85 procedures with at least one positive culture, up to 84.7% showed differing microbiological profiles between both compartments, highlighting the added diagnostic value of intraoperative cultures. This challenges the reliability of preoperative cultures as the sole risk indicator and may lead to unnecessary antimicrobial treatments targeting organisms that are not clinically relevant [26,27].

Our results highlight the limited concordance between bladder urine and renal pelvic cultures, with over 80% of positive cases showing discordant microbiological profiles. This finding questions the reliability of preoperative bladder urine cultures as the sole guide for prophylactic antibiotic selection. Previous studies have emphasized the discrepancy: Mariappan et al. demonstrated that stone and renal pelvic cultures more accurately predicted postoperative sepsis than bladder urine cultures, while Nevo et al. reported similar findings in patients with ureteral stents [26,27].

The potential presence of bacteria within the stone matrix or the renal collecting system, not detected in bladder urine, may explain this divergence. Collecting intraoperative samples, when feasible, provides valuable microbiological data to optimize postoperative management, particularly in patients at high risk of infection or with recurrent sepsis [26,27].

Although not routinely performed in all centers, our experience supports its selective use to improve microbiological precision and therapeutic decisions.

Another relevant aspect is operative time. In our series, the median duration was 100 min (IQR 35–180), in line with previous reports. Several cohort studies and meta-analyses have shown that longer operative times—particularly beyond 90 to 100 min—are associated with a higher risk of postoperative infectious complications, including sepsis. These findings underscore the impact of surgical duration on infection development, particularly in patients with elevated baseline risk [7,8].

This study has several limitations that should be acknowledged. First, its retrospective and single-center design inherently carries a risk of selection and information bias. Although the protocol was consistently applied, we cannot exclude the possibility of unmeasured confounders influencing the results. Second, while infectious outcomes were clearly defined, microbiological reassessment beyond hospitalization was not routinely performed unless clinically indicated, limiting our ability to assess ecological impact or late resistance patterns.

Third, the microbiological heterogeneity of stone disease may have influenced culture results, particularly in discordant cases. Approximately 10% of patients underwent repeat procedures, which could have introduced bias in subgroup analyses. Finally, although prolonged operative time has been associated with increased infectious risk in previous studies—such as those by Zhou et al. and Puia et al.—we did not observe this association in our cohort. This may reflect the multifactorial nature of surgical complexity and suggests that operative time alone may be an insufficient surrogate for procedural risk, warranting more detailed analysis in future studies [7,8].

Future studies should aim to validate our findings in larger, prospective multicenter cohorts that include long-term follow-up and cost-effectiveness analyses. Stratifying patients by infectious risk, stone burden, and microbiological profile could allow for more precise prophylactic algorithms and individualized surgical planning. In addition, incorporating more intraoperative data such as number of access tracts, degree of stone fragmentation, or bleeding may help refine the evaluation of surgical complexity as a risk factor. Finally, the role of renal pelvic or stone cultures in guiding targeted therapy deserves further exploration, particularly in patients with discordant bladder urine findings or recurrent infections.

## 5. Conclusions

The implementation of a single-dose, culture-guided antibiotic prophylaxis protocol at our center was not associated with an increased rate of infectious complications following percutaneous nephrolithotomy, even in high-risk patients. While these findings are encouraging, further prospective studies are needed to validate their generalizability.

Our results support the rationale behind antimicrobial stewardship programs, suggesting that individualized prophylaxis based on microbiological data can be both safe and efficient. This approach reinforces the importance of careful preoperative urine culture assessment and antibiotic selection tailored to each patient.

## Figures and Tables

**Table 1:** Baseline patient characteristics.

Category	Data	Value n (%) or Mean (SD)
Age		57.06 (14.6)
BMI		27.04 (4.96)
Gender	Male	87 (46.5)
	Female	100 (53.5)
Diabetes mellitus	Type 1	1 (0.5)
	Type 2	33 (17.6)
History of septic risk conditions	Immunosuppressive disease	1 (0.5)
	Active oncological disease	2 (1.1)
	Chemotherapy	3 (1.6)
	Previous febrile UTI/sepsis	35 (18.7)
	Multiple risk factors above	1 (0.5)
	Other	4 (2.1)
History of prior surgery on the target stone	ESWL	5 (2.7)
	URS	4 (2.1)
	RIRS	9 (4.8)
	Multiple procedures	13 (7.0)
Presence of urinary drainage device prior to surgery	Double-J stent	48 (25.7)
	Nephrostomy tube	8 (4.3)
	Both (DJ + NPC)	18 (9.6)
Preoperative urine culture	Sterile	100 (58.1)
	Positive	60 (34.8)
	Contaminated	12 (7)

**Table 2:** Microbiological isolates in patients with positive urine cultures (preoperative and renal puncture).

Microorganism	Preoperative Urine Culture n (%)	Renal Puncture Culture n (%)	Change in Microbiological Isolate n (%) *
*E. coli*	23 (37.7)	9 (37.5)	26 (81.2)
*Proteus* spp.	11 (18.03)	7 (29.17)	15 (83.3)
*Klebsiella* spp.	7 (11.48)	1 (4.17)	7 (87.5)
*Enterococcus* spp.	6 (9.84)	3 (12.5)	7 (77.8)
Other **	14 (22.95)	4 (16.7)	17 (94.5)
Total	61 (100)	24 (100)	72 (84.7)

* Change was defined as either a mismatch in isolated microorganisms between the preoperative urine culture and the renal puncture culture, or the presence of a positive preoperative culture with a sterile intraoperative sample. ** Other: *Aerococcus*, *Citrobacter koseri*, *Candida albicans*, and mixed anaerobic flora.

**Table 3:** Infectious complications in high-risk patients.

		OR (95% CI)			
Outcome	Rate (%)	Positive UC	DM	Prior Drainage	Guy’s IV
Fever	8.7	0.98 (0.53–1.80)	0.71 (0.31–1.66)	1.31 (0.67–2.55)	1.01 (0.36–2.78)
Sepsis	3.2	1.09 (0.40–2.96)	8.8 (0.8–87)	0.77 (0.42–1.38)	**
Postoperative fever/sepsis ***	11.9	1.27 (0.33–4.91)	2.52 (0.67–9.37)	1.03 (0.28–3.72)	0.69 (0.13–3.62)

** No events in control group. *** Multivariable analysis for composite infectious complications outcome.

## Data Availability

Source data are available for review upon request.

## Abbreviations

The following abbreviations are used in this manuscript:

PCNL	Percutaneous Nephrolithotomy
UTI	Urinary Tract Infection
ASPs	Antimicrobial Stewardship Programs
EAU	European Association of Urology
AUA	American Urological Association
SEIMC	Spanish Society of Infectious Diseases and Clinical Microbiology (Sociedad Española de Enfermedades Infecciosas y Microbiología Clínica)
AEC	Spanish Association of Surgeons (Asociación Española de Cirujanos)
SOFA	Sequential Organ Failure Assessment
DJ	Double-J Stent
NPC	Nephrostomy Catheter
ESWL	Extracorporeal Shock Wave Lithotripsy
